# Study on the correlation between energy availability and subclinical menstrual disorders

**DOI:** 10.3389/fnut.2024.1479254

**Published:** 2024-11-29

**Authors:** Zhou Bingzheng, Jin Zhuo, Wang Qihao, Bai Lunhao

**Affiliations:** ^1^Department of Orthopedics and Sports Medicine, Shengjing Hospital of China Medical University, Shenyang, Liaoning, China; ^2^Research Center for Universal Health, School of Public Health of China Medical University, Shenyang, Liaoning, China; ^3^Center of Reproductive Medicine, Department of Obstetrics and Gynecology, Shengjing Hospital of China Medical University, Shenyang, Liaoning, China

**Keywords:** energy availability, subclinical menstrual disorders, luteal phase defects, low energy availability, anovulation

## Abstract

**Background:**

There are few studies on the correlation between energy availability (EA) and subclinical menstrual disorders (SMD) in female athletes. This study aims to explore the differences in EA between female athletes with eumenorrhea and those with SMD, and the correlation between EA and the occurrence of SMD.

**Methods:**

Luteal phase defect (LPD) and anovulation were defined as SMD. Fifty-six adult female college athletes with regular menstrual cycles and no clinical symptoms of menstrual disorders (MD) were selected as subjects. The EA of the subjects was monitored, and SMD were identified. The EA was compared between the two groups, and the correlation between EA and the occurrence of SMD was analyzed.

**Results:**

Nineteen subjects were identified as LPD, and 7 subjects were identified as anovulation. The occurrence of SMD was 46.4%. The EA of subjects with SMD was lower than that of subjects with eumenorrhea (*t* = 3.165, *p* = 0.003), and EA was negatively correlated with the occurrence of SMD (*r* = −0.396, *p* = 0.000).

**Conclusion:**

There were differences in EA between female college athletes with eumenorrhea and SMD. EA was negatively correlated with the occurrence of SMD.

## 1 Introduction

Energy balance is crucial for athletes to maintain health and achieve excellent sports performance. Once athletes have insufficient energy intake (EI) or/and excessive exercise energy expenditure (EEE), the body lacks sufficient energy to maintain optimal health. In medicine, the condition in which energy imbalance leads to impaired physiological function of multiple organ systems is defined as Relative Energy Deficiency in Sport (RED-S), which expands the previous diagnostic of the Female Athlete Triad (FAT) (1, 2).

The importance of EA in maintaining reproductive health has been confirmed (3, 4). Loucks (4) found that when the EA of healthy women was below 30 kcal/kg FFM for 5 consecutive days, the pulsatile release of luteinizing hormone began to decrease. The change might lead to abnormal secretion of estrogen and progesterone in the ovaries, ultimately resulting in MD (5, 6). Since Lucas proposed the hypothesis of the EA threshold for inducing MD, researchers have conducted corresponding validations (7–9). Lieberman (7) and Williams (8) found that in sedentary healthy females, controlling EA below the threshold did not induce MD in all subjects, but showed a difference in EA between the group with eumenorrhea and MD. Reed (9) divided the subjects into eumenorrhea group, SMD group, and MD group based on reproductive health. The results of the study found that EA was different in the group with eumenorrhea and MD, but not in the group with eumenorrhea and SMD. Notably, Lieberman and Williams’s study defined LPD, anovulation, and oligomenorrhea as MD (7, 8). Reed’s study defined inconsistent ovulation, anovulation, and oligomenorrhea as SMD, and amenorrhea as MD (9).

Currently, the definition of MD is relatively broad. According to the severity of its continuous occurrence, MD can be divided into LPD, anovulation, oligomenorrhea, and amenorrhea. LPD and anovulation are considered subclinical states of MD and can usually quickly return to eumenorrhea by supplementing energy or reducing exercise consumption (10–12). Therefore, it is necessary to explore whether EA is different between eumenorrhea and SMD based on the renewed understanding of MD. Unlike Reed’s study, this study defined LPD and anovulation as SMD.

## 2 Materials and methods

### 2.1 Subjects

**Figure 1:**
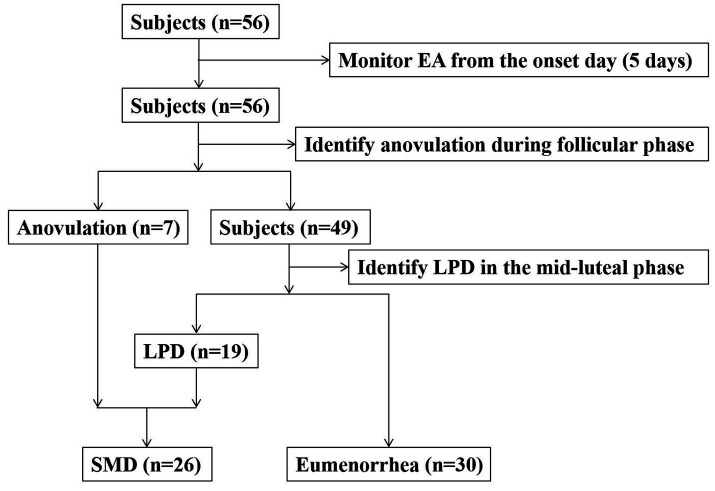
Flow chart of this study.

We recruited subjects from the women’s soccer, basketball, volleyball, and track teams of a university. The inclusion criteria for subjects were as follows: age ≥ 18 years, no history of pregnancy, no history of eating disorders, no use of contraceptive medications in the 12 months prior to the study, and a self-reported menstrual cycle stability (26–35 days). Subjects who experienced oligomenorrhea or amenorrhea after the start of the study would be withdrawn. The research process is shown in Figure 1. Finally, 56 female college athletes who met the criteria voluntarily participated in this study. All subjects received written and verbal information about the study prior to participation and signed a written informed consent form. This study was approved by the institutional review board and strictly adhered to the relevant principles of the Declaration of Helsinki.

### 2.2 Test of EI, EEE and demographic parameters

A menstrual calendar was used to record the onset day and days of the subjects’ menstrual cycle. On the onset day of the menstrual cycle, EI and EEE were monitored for 5 consecutive days. Dietary nutrition analysis software (Health Technology, Shanghai, China) was used to analyze the data. The subjects’ training diaries were recorded, including exercise type and exercise time, and the exercise energy consumption was analyzed by calculating metabolic equivalent (13).

A body composition analyzer (Omron, BCA-1A, OUQI Biotech, Shanghai, China) was used to test demographic parameters. On the morning of the 7th day of the menstrual cycle, the demographic parameters of the subjects were tested. The subjects should avoid strenuous exercise within 24 h before the test, fast at night, and empty their bowels and bladder on the morning of the next day before the test. Tests were conducted on the four limbs with an 8-electrode electrical impedance device using a 0.8-ma current at 5, 50, 250, and 500 kHz. Four electrodes were attached to the bilateral palms and thumbs, and the other 4 electrodes were attached to the anterior and posterior soles bilaterally. The test process strictly followed the instructions. EA was calculated as EA = Daily (EI-EEE) /FFM.

### 2.3 Identification of LDP and anovulation

On the day after the bleeding stopped, the ovulation prediction kit (Diagnostic Kit for luteinizing Hormone Colloidal Gold, ACON Biotech, Hangzhou, China) was used to monitor the subjects’ morning urine samples every day until the end of the ovulation phase. Subjects took photos of the test using their smartphones and sent the picture to the study manager, who verified whether the test was positive or not. A negative result was defined as anovulation and the next stage of progesterone monitoring was terminated.

After ovulation, the median day of the luteal phase was calculated and defined as the mid-luteal phase. A fasting blood sample (8 mL) was obtained in the morning of the day corresponding to the mid-luteal phase. The blood samples were transferred to a 10 mL polypropylene tube and then sent to the laboratory. The blood samples were centrifuged at 1500 g for 15 min, and the serum was separated for detection. The detection instrument was the automatic particle chemiluminescence immune analyzer (UnicelTM DXI800Accsss, Beckman Coulter, Inc., United States), and the detection kit was purchased from the company. LPD was defined as the progesterone concentration < 5.12 ng/mL.

### 2.4 Statistical analysis

Continuous variables were expressed as mean ± SD and categorical variables were presented as counts with percentages. Shapiro–Wilk test was used to assess the normality of the data. T-test was used to compare continuous variables. Point-biserial correlation was used to analyze correlations. Data were processed using the SPSS 16.0 (Chicago, IL, United States), and *p* < 0.05 was considered statistically significant.

## 3 Results

None of the 56 enrolled subjects demonstrated oligomenorrhea or amenorrhea. Nineteen (33.9%) subjects were LPD, and 7 (12.5%) subjects were anovulation. The occurrence of SMD was 46.4%. Compared with the eumenorrhea group, the SMD group had lower fat mass, percentage body fat (PBF), EEE, and EA (*p* < 0.05). There were no significant differences in age, timing of menarche, cycle length, stature, body mass, BMI, lean body mass, and EI between the two groups (*p* > 0.05; Table 1).

**Table 1:** Comparison of variables between the two groups.

Variables	Eumenorrhea	SMD	*t*	*p*
Age (year)	22.3 ± 2.0	21.6 ± 2.0	1.299	0.200
Timing of menarche (year)	12.5 ± 1.3	11.8 ± 1.6	1.594	0.117
Cycle length (day)	28.8 ± 1.7	28.0 ± 1.7	1.831	0.073
Stature (cm)	175.9 ± 9.6	174.3 ± 10.3	0.610	0.544
Body mass (kg)	64.4 ± 9.2	61.3 ± 8.4	1.305	0.197
BMI (kg/m^2^)	20.8 ± 2.3	20.2 ± 2.3	0.999	0.322
Fat mass (kg)	11.5 ± 3.6	8.1 ± 1.2	4.551	0.000*
Lean body mass (kg)	52.9 ± 8.8	53.2 ± 8.1	4.015	0.903
PBF (%)	18.0 ± 5.4	13.4 ± 2.3	−0.122	0.000*
EI (Kcal)	2760.1 ± 379.8	2802.2 ± 333.3	−0.436	0.664
EEE (Kcal)	911.87 ± 252.8	1196.8 ± 212.1	−4.527	0.000*
EA (kcal/kg FFM/day)	34.7 ± 6.8	30.2 ± 2.2	3.165	0.003*

*Statistically significant difference (*p* < 0.05).

In correlation analysis, age, timing of menarche, cycle length, stature, body mass, BMI, lean body mass, and EI were not associated with the occurrence of SMD (*p* > 0.05). Fat mass, PBF, EEE, and EA were correlated with the occurrence of SMD (*p* < 0.05).

## 4 Discussion

This study found that LPD and anovulation had a relatively high occurrence rate (46.4%), which was consistent with the previous studies (50–55%) (14, 15). In terms of the identification methods for LPD and anovulation, this study adopted a combination of menstrual calendars, urine pregnancy tests, and serum progesterone tests. Early studies mainly relied on the measurement of daily hormone levels to identify LPD and anovulation. However, the frequency of specimen collection and its economic cost became the main challenges of such studies (16, 17). Recent studies have shown that using menstrual calendars, urine pregnancy tests, and serum progesterone tests can effectively identify LPD and anovulation with the lowest frequency of specimen collection and economic cost (18, 19).

Currently, in the classification of MD, LPD and anovulation usually have regular menstrual cycles and lack obvious clinical symptoms compared to oligomenorrhea and amenorrhea. This characteristic makes it difficult to identify LPD and anovulation by questionnaire surveys (16, 17). Anovulation refers to the phenomenon where the ovaries fail to form mature follicles and thus cannot ovulate. LPD refers to insufficient progesterone secretion during the luteal phase or the premature regression of the corpus luteum (16, 17). In terms of identification methodology, ovulation prediction kits are non-invasive and cost-effective tools that can indirectly identify anovulation. For LPD, invasive serum progesterone test is still considered the gold standard method for current identification (16, 17). Currently, there is no consensus on the minimum progesterone level required for reliable diagnosis of LPD (16 or 9.54 nmol/L) (20–24). Relevant studies recommend that when performing a single measurement, the progesterone concentration should be set at a conservative limit of at least 16 nmol/L (16). Therefore, in this study, the progesterone concentration < 16 nmol/L (5.12 ng/mL) was defined as LPD.

In the relevant study on EA and MD, Lieberman and Williams’s study defined LPD, anovulation, and oligomenorrhea as MD, and found that EA was different between subjects with eumenorrhea and those with MD (7, 8). Reed’s study divided the subjects into the eumenorrhea group, oligomenorrhea group, and amenorrhea group according to reproductive health, and then further divided the eumenorrhea group into the normal ovulation group, inconsistent ovulation group, and anovulation group. In Reed’s study, the inconsistent ovulation group, anovulation group, and oligomenorrhea group were defined as SMD. The results found that there was no difference in EA between the normal ovulation group and the SMD group (9). Notably, Reed’s study classified the oligomenorrhea group as SMD. Meanwhile, the inconsistent ovulation group was also defined as SMD. Currently, in the definition of exercise-induced MD, inconsistent ovulation has not been included in the corresponding category (10–12). Unlike the above studies, this study only defined LPD and anovulation as SMD, and the results showed that EA was different between eumenorrhea and SMD.

Energy availability is the utilization of energy to maintain the normal physiological function of the human body. When the body is in low energy availability (LEA; < 30 kcal/kg FFM/day), the body will sacrifice the energy supply for reproductive health in order to restore energy balance (25). Therefore, EA < 30 kcal/kg FFM/day is also internationally defined as LEA (26, 27). Currently, although the EA threshold for MD has not been confirmed, corresponding studies have shown that there is a dose–response relationship between EA and the occurrence of MD (7–9). This study found that the average EA value in the SMD group was 30.2 kcal/kg FFM/day, which was higher than the defined value of LEA. LPD and anovulation are considered subclinical states of MD. We believe that conducting study on such populations can better prevent FAT and RED-S based on EA monitoring. Additionally, the specific values of LEA may be redefined in the future.

Currently, due to the broad definition of clinical stages of MD, including different clinical stages may lead to contradictory conclusions. Therefore, it is necessary to clarify the clinical stages of MD in the subjects. In addition, there is still a lack of unified EA calculation methods internationally. The calculation of EA needs to involve both EI and EEE. EI can be evaluated through methods such as food weighing or dietary records, while EEE can be evaluated through methods such as exercise logs, metabolic energy expenditure, or data collected from wearable devices (25–27). Different methods may lead to contradictory conclusions. Therefore, it is necessary to establish internationally unified standards in methodology in the future.

## 5 Limitation

The sample size of this study was small. According to the clinical status of MD, this study lacked a comparison of subjects with oligomenorrhea and amenorrhea. In terms of EI and EEE collection, self-reporting might result in underreporting (28).

## 6 Conclusion

LPD and anovulation had a higher occurrence in female college athletes with regular menstrual cycles and no clinical symptoms of MD. EA was different between eumenorrhea and SMD, and was negatively associated with the occurrence of SMD.

## Data Availability

The original contributions presented in the study are included in the article/Supplementary material, further inquiries can be directed to the corresponding author.
