# Supplementary material for: Study on the correlation between energy availability and subclinical menstrual disorders
Source: Front Nutr. 2024 Nov 29;11:1479254. doi: 10.3389/fnut.2024.1479254 (PMC11637835; doi:10.3389/fnut.2024.1479254)
Supplement: Supplementary file 1 [file Table_1.DOCX]

Supplementary Material

# Supplementary Data

| NO | SPORTS | Age (year) | Timing of menarche  (year) | Cycle length (day) | Stature (cm) | Body mass (kg) | BMI (kg/m^2^) | Fat mass (kg) | PBF (%) | Lean body mass (kg) | EI (Kcal) | EEE (Kcal) | EA (kcal/kg FFM/day) | Anovulation | LPD  (Progesterone concentration (ng/ml)) |
| --- | --- | --- | --- | --- | --- | --- | --- | --- | --- | --- | --- | --- | --- | --- | --- |
| 1 | SOCCER | 22 | 11 | 29 | 178 | 76.1 | 24.0 | 12.8 | 16.8 | 63.3 | 3178 | 1359 | 28.7 | No | No (5.76) |
| 2 | SOCCER | 21 | 13 | 27 | 174 | 50.2 | 16.6 | 5.8 | 11.6 | 44.4 | 2471 | 1154 | 29.7 | Yes | - |
| 3 | SOCCER | 21 | 12 | 29 | 171 | 51.7 | 17.7 | 6.5 | 12.6 | 45.2 | 2508 | 1008 | 33.2 | No | No (5.44) |
| 4 | SOCCER | 21 | 12 | 27 | 170 | 63.4 | 21.9 | 9.2 | 14.5 | 54.2 | 2859 | 1286 | 29.0 | No | Yes (4.80) |
| 5 | SOCCER | 24 | 11 | 29 | 173 | 66.2 | 22.1 | 7.5 | 11.3 | 58.7 | 3108 | 1552 | 26.5 | No | Yes (4.48) |
| 6 | SOCCER | 24 | 12 | 27 | 161 | 51.6 | 19.9 | 5.2 | 10.1 | 46.4 | 2778 | 1378 | 30.2 | Yes | - |
| 7 | SOCCER | 22 | 14 | 29 | 164 | 62.5 | 23.2 | 8.8 | 14.1 | 53.7 | 2714 | 927 | 33.3 | No | No (5.76) |
| 8 | SOCCER | 23 | 11 | 29 | 175 | 56.1 | 18.3 | 7.9 | 14.1 | 48.2 | 2506 | 1133 | 28.5 | No | Yes (4.16) |
| 9 | SOCCER | 23 | 11 | 32 | 175 | 63.7 | 20.8 | 6.3 | 9.9 | 57.4 | 3113 | 1052 | 35.9 | No | No (5.52) |
| 10 | SOCCER | 22 | 15 | 30 | 167 | 62.5 | 22.4 | 9.8 | 15.7 | 52.7 | 2630 | 1195 | 27.2 | No | Yes (3.72) |
| 11 | SOCCER | 21 | 12 | 27 | 167 | 51.3 | 18.4 | 6.4 | 12.5 | 44.9 | 2893 | 1471 | 31.7 | No | Yes (4.98) |
| 12 | SOCCER | 21 | 13 | 29 | 163 | 47.3 | 17.8 | 7.4 | 15.6 | 39.9 | 2250 | 888 | 34.1 | No | No (5.23) |
| 13 | SOCCER | 21 | 12 | 32 | 165 | 59.2 | 21.7 | 8.3 | 14.0 | 50.9 | 2736 | 1283 | 28.5 | No | Yes (2.88) |
| 14 | SOCCER | 20 | 13 | 29 | 161 | 49.8 | 19.2 | 8.8 | 17.7 | 41.0 | 2139 | 925 | 29.6 | No | Yes (3.94) |
| 15 | SOCCER | 25 | 14 | 28 | 161 | 64.5 | 24.9 | 9.7 | 15.0 | 54.8 | 2947 | 1482 | 26.7 | No | Yes (4.16) |
| 16 | SOCCER | 22 | 11 | 26 | 164 | 59.3 | 22.0 | 9.8 | 16.5 | 49.5 | 2634 | 1209 | 28.8 | No | Yes (4.48) |
| 17 | SOCCER | 21 | 14 | 30 | 160 | 65.4 | 25.5 | 11.6 | 17.7 | 53.8 | 2653 | 1127 | 28.4 | No | No (5.54) |
| 18 | SOCCER | 23 | 13 | 29 | 175 | 71.2 | 23.2 | 13.5 | 19.0 | 57.7 | 3060 | 646 | 41.8 | No | No (6.08) |
| 19 | SOCCER | 26 | 13 | 30 | 171 | 66.2 | 22.6 | 8.5 | 12.8 | 57.7 | 3216 | 608 | 45.2 | No | No (6.41) |
| 20 | SOCCER | 24 | 11 | 31 | 165 | 57.4 | 21.1 | 7.2 | 12.5 | 50.2 | 2576 | 956 | 32.3 | No | No (5.47) |
| 21 | SOCCER | 22 | 13 | 28 | 170 | 67.8 | 23.5 | 8.3 | 12.2 | 59.5 | 2872 | 958 | 32.2 | No | No (6.02) |
| 22 | BASKETBALL | 21 | 15 | 30 | 200 | 90.1 | 22.5 | 19.4 | 21.5 | 70.7 | 3431 | 926 | 35.4 | No | No (6.11) |
| 23 | BASKETBALL | 21 | 14 | 28 | 178 | 70.4 | 22.2 | 8.6 | 12.2 | 61.8 | 2934 | 1176 | 28.4 | Yes | - |
| 24 | BASKETBALL | 22 | 13 | 26 | 180 | 73.6 | 22.7 | 9.9 | 13.5 | 63.7 | 3077 | 1297 | 27.9 | No | Yes (3.84) |
| 25 | BASKETBALL | 21 | 11 | 26 | 174 | 61.2 | 20.2 | 7.6 | 12.4 | 53.6 | 2718 | 1208 | 28.2 | No | Yes (3.52) |
| 26 | BASKETBALL | 23 | 10 | 27 | 185 | 79.7 | 23.3 | 7.6 | 9.5 | 72.1 | 3187 | 1108 | 28.8 | Yes | - |
| 27 | BASKETBALL | 22 | 14 | 32 | 187 | 82.3 | 23.5 | 9.8 | 11.9 | 72.5 | 3406 | 852 | 35.2 | No | No (5.76) |
| 28 | BASKETBALL | 26 | 12 | 30 | 175 | 67.8 | 22.1 | 9.1 | 13.4 | 58.7 | 3263 | 1756 | 25.7 | No | Yes (4.16) |
| 29 | BASKETBALL | 23 | 10 | 28 | 185 | 69.2 | 20.2 | 11.1 | 16.0 | 58.1 | 3172 | 1172 | 34.4 | No | No (5.44) |
| 30 | BASKETBALL | 21 | 12 | 30 | 171 | 66.4 | 22.7 | 9.3 | 14.0 | 57.1 | 3091 | 556 | 44.4 | No | No (5.28) |
| 31 | BASKETBALL | 23 | 11 | 28 | 180 | 54.5 | 16.8 | 7.2 | 13.2 | 47.3 | 2653 | 1368 | 27.2 | No | Yes (3.20) |
| 32 | BASKETBALL | 22 | 14 | 30 | 177 | 64.3 | 20.5 | 10.6 | 16.5 | 53.7 | 2877 | 567 | 43.0 | No | No (5.32) |
| 33 | BASKETBALL | 22 | 12 | 27 | 181 | 74.1 | 22.6 | 15.9 | 21.5 | 58.2 | 3049 | 1439 | 27.7 | No | No (5.46) |
| 34 | VOLLEYBALL | 20 | 12 | 28 | 190 | 72.4 | 20.1 | 8.3 | 11.5 | 64.1 | 3125 | 1058 | 32.2 | No | Yes (2.88) |
| 35 | VOLLEYBALL | 19 | 10 | 29 | 187 | 72.7 | 20.8 | 7.6 | 10.5 | 65.1 | 3374 | 1503 | 28.7 | Yes | - |
| 36 | VOLLEYBALL | 23 | 15 | 28 | 189 | 66.3 | 18.6 | 14.3 | 21.6 | 52 | 2765 | 838 | 37.1 | No | No (6.40) |
| 37 | VOLLEYBALL | 25 | 12 | 30 | 183 | 64.2 | 19.2 | 13.6 | 21.2 | 50.6 | 2498 | 850 | 32.6 | No | No (6.08) |
| 38 | VOLLEYBALL | 20 | 13 | 26 | 194 | 73.1 | 19.4 | 6.5 | 8.9 | 66.6 | 3338 | 663 | 40.2 | No | No (5.22) |
| 39 | VOLLEYBALL | 20 | 12 | 31 | 190 | 64.7 | 17.9 | 9.1 | 14.1 | 55.6 | 2956 | 1374 | 28.5 | No | Yes (3.53) |
| 40 | VOLLEYBALL | 18 | 14 | 25 | 193 | 64.6 | 17.3 | 7.2 | 11.2 | 57.4 | 3255 | 1588 | 29.0 | No | Yes (3.84) |
| 41 | VOLLEYBALL | 18 | 11 | 28 | 188 | 64.3 | 18.2 | 13.0 | 20.2 | 51.3 | 2437 | 1008 | 27.9 | No | No (6.08) |
| 42 | VOLLEYBALL | 22 | 9 | 28 | 191 | 61.2 | 16.8 | 7.6 | 12.4 | 53.6 | 2755 | 1265 | 27.8 | No | Yes (4.11) |
| 43 | VOLLEYBALL | 20 | 12 | 27 | 182 | 60.1 | 18.1 | 18.2 | 30.3 | 41.9 | 2338 | 725 | 38.5 | No | No (8.02) |
| 44 | VOLLEYBALL | 19 | 10 | 25 | 177 | 63.0 | 20.1 | 7.4 | 11.8 | 55.6 | 2838 | 1438 | 25.2 | Yes | - |
| 45 | VOLLEYBALL | 24 | 13 | 29 | 175 | 58.6 | 19.1 | 13.4 | 22.9 | 45.2 | 2617 | 563 | 45.4 | No | No (6.42) |
| 46 | VOLLEYBALL | 20 | 14 | 32 | 176 | 55.9 | 18.0 | 13.7 | 24.5 | 42.2 | 2006 | 1325 | 16.1 | No | No (7.03) |
| 47 | VOLLEYBALL | 19 | 12 | 26 | 167 | 61.1 | 21.9 | 10.9 | 17.8 | 50.2 | 2518 | 657 | 37.1 | No | No (5.76) |
| 48 | VOLLEYBALL | 25 | 11 | 27 | 172 | 60.3 | 20.4 | 10.5 | 17.4 | 49.8 | 2572 | 705 | 37.5 | No | No (5.44) |
| 49 | VOLLEYBALL | 20 | 9 | 28 | 163 | 49.8 | 18.7 | 7.4 | 14.9 | 42.4 | 2377 | 1482 | 21.1 | No | Yes (3.20) |
| 50 | Track and field | 22 | 12 | 26 | 181 | 60.2 | 18.4 | 17.4 | 29.0 | 42.8 | 2534 | 796 | 40.6 | No | No (7.68) |
| 51 | Track and field | 23 | 12 | 27 | 171 | 61.7 | 21.1 | 16.7 | 27.1 | 45 | 2562 | 1058 | 33.4 | No | No (6.97) |
| 52 | Track and field | 19 | 13 | 28 | 170 | 54.6 | 18.9 | 8.4 | 15.4 | 46.2 | 2609 | 1242 | 29.6 | No | Yes (2.65) |
| 53 | Track and field | 22 | 13 | 28 | 163 | 44.3 | 16.7 | 9.5 | 21.4 | 34.8 | 2062 | 1404 | 18.9 | No | No (6.08) |
| 54 | Track and field | 25 | 10 | 28 | 173 | 62.8 | 21.0 | 8.9 | 14.2 | 53.9 | 2748 | 766 | 36.8 | No | No (5.43) |
| 55 | Track and field | 24 | 13 | 29 | 161 | 49.5 | 19.1 | 9.2 | 18.6 | 40.3 | 2036 | 785 | 31.0 | Yes | - |
| 56 | Track and field | 26 | 13 | 29 | 171 | 63.5 | 21.7 | 10.8 | 17.0 | 52.7 | 2647 | 957 | 32.1 | No | No (5.12) |
